# Spatial regulation of ribosomal protein gene expression revealed by spatial transcriptomic analysis in the water fern *Ceratopteris richardii*

**DOI:** 10.3389/fpls.2025.1728120

**Published:** 2026-01-19

**Authors:** Jin Han, Gui-Sheng Li

**Affiliations:** Laboratory of Plant Resource Conservation and Utilization, Jishou University, Jishou, China

**Keywords:** *Ceratopteris richardii*, *in situ* hybridization, leaf primordia, ribosomal protein genes, spatial transcriptome, vasculature

## Abstract

Spatial transcriptomics is proving to be a powerful tool in elucidating plant development complexities. A study on the water fern *Ceratopteris richardii* applied this technology to sporophyte development, identifying 11 distinct spot clusters that mapped to leaves at various stages and probably leaf primordia and the shoot apical meristem (SAM), the stem, the vasculature, and scales/trichomes. Several transcription factors were identified—from the *ARF*, *GRF*, *AP2*, and *bZIP* families—as marker genes for these clusters. Functional enrichment analysis of marker genes identified biologically important processes, such as chloroplast-related functions in advanced leaves and DNA helicase activity in mitotic tissues. The pervasive enrichment of ribosome-related functions in nearly all tissue domains was unexpected. Furthermore, ribosomal protein (RP) genes were differentially expressed between tissues, marking single or multiple tissues. Paralogs encoding the same type of RP also differed in expression patterns from one another, with certain members not expressed or uniformly expressed. *in situ* hybridization showed that RP genes were generally expressed in the SAM, leaf primordia, and the vasculature.

## Introduction

Ribosomes comprise molecules of protein and RNA, providing translation sites for cells. The hypothesis of one gene, one ribosome, and one protein has been postulated, suggesting that one cell contains thousands of different ribosomes and each produces a certain type of protein. Although the idea that one ribosome entity can produce all types of proteins still prevails in current textbooks, ribosomal protein (RP) genes have been found specifically expressed in terms of developmental stages, structure tissues, and single cells, which sometimes is related to certain phenotypes. For example, ribosomes containing RPL39L rather than the paralogous RPL39 create a hydrophobic patch that can facilitate co-translational folding of a subset of germ-specific proteins in mice (Li et al., 2022; Banerjee et al., 2024). Indeed, the active translation of ribosomes can bear the lack of one or more core proteins in mouse embryonic stem cells (Shi et al., 2017). Second, rRNA copies contain ample nucleotide polymorphism in human and mice (Parks et al., 2018), and the variants can preferentially bind certain mRNAs in zebrafish (Locati et al., 2017) or specialize ribosomes for programed frameshifting in *Saccharomyces cerevisiae* (Kiparisov et al., 2005). Third, chemical modification is also diverse among core proteins and rRNAs, and ribosome-associated protein composition can change, thus leading to ribosomal heterogeneity (Joo et al., 2022; Cunningham et al., 2023; Park et al., 2024). Ribosome assembly may be very dynamic in response to stimuli; thus, a certain ribosome type may exist in a single cell or a particular subcellular region. Specialized ribosomes can recognize certain cis-regulatory elements that reside in the 5’ UTR of mRNAs for expression regulation (Leppek et al., 2018). For example, several *Hox* mRNAs contain the internal ribosome entry site (IRES) that depends on RPL38/eL38 for their function in the 5’ UTR, and removal of these IRESes ablates Hox protein but not mRNA biogenesis in mice (Xue et al., 2015). Thus, there may be one mRNA regulon-one ribosome-one protein network, where specific phenotypes rather than a global translation depression arise when components of ribosomes are destroyed (Genuth and Barna, 2018). Nevertheless, RPL3L depletion affects neither translational efficiency nor ribosome affinity toward a subset of transcripts but increases ribosome-mitochondrion interaction for generating more ATP in cardiomyocytes (Milenkovic et al., 2023). Remarkably, ribosomal homeostasis has recently been proposed for ribosomal effects on plant development (Martinez et al., 2025).

Spatial transcriptomics aims to generate a gene expression matrix, where the capturing-based technology can provide a gene position by anchoring tissue sections onto a slide and ligating tissue poly-adenylated RNA to barcoded molecule labels. Using slides consisting of ~1000 positioned spots (100 μm spot diameter with 200 μm center-to-center distance), the RNA captured by spots can provide an unbiased investigation for a section transcriptome (Ståhl et al., 2016). The Visium product released by 10X Genomics can provide a satisfactory resolution (55 μm spot diameter with center-to-center distance of 100 μm) and sensitivity (~10000 RNA molecules per spot). Spot clustering can define sets of spots possessing similar transcriptomes after matrix normalization, which can reveal the tissue composition of sections, for example, the stem, meristems, sepals, and petals residing in a single section (Giacomello et al., 2017). Numerous methods have been developed for the identification of spatially variable genes (SVGs) that exhibit non-random and informative spatial patterns, promoting spatial domain recognition and marker gene determination (Yan et al., 2025). Spatial transcriptomics can be used to characterize cells with the help of single-cell RNA-seq when the spot is beyond cell resolution or to characterize subcellular compartments with the local density of each RNA species when the spot is below cell resolution (Rao et al., 2021). Spatial transcriptomics has been widely employed in plant development research (Yin et al., 2023).

Ferns are the only sister group of seed plants with a divergence time of ~400 million years (Pryer et al., 2004), and the genus *Ceratopteris* is frequently used in fern biology exploration. For example, spore germination (Kamachi et al., 2004; Cannon et al., 2023) and gametophyte development (Bartz and Gola, 2018; Withers et al., 2023; Renninger et al., 2025; Xie et al., 2025) have been extensively studied in *C. richardii*. Fens possess a free-living sporophyte for the first time during evolution of land plants. A handful of genes related to sporophyte development have recently been investigated. *Class 1 Knotted1-like homeobox* (*KNOXI*) genes are expressed in the shoot apical meristem (SAM), leaf primordia, and marginal regions of fern leaves, as they expressed in angiosperms possessing compound leaves (Sano et al., 2005; Vasco and Ambrose, 2020). These genes are also expressed in the leaf apical meristem and pinnae primordia, which correlates with delayed determinacy of fern leaves (Cruz et al., 2020). *KANADI* (*KAN*) genes, as well as *Class III homeodomain-leucine zipper* (*HD-Zip III*) genes, are expressed as homologs in flowering plants do for determination of leaf dorsoventrality (Vasco et al., 2016; Zumajo-Cardona et al., 2019). RNAi of a *WUSCHEL-RELATED HOMEOBOX* (*WOX*) gene, namely *CrWOXB*, can reduce the number of leaves, pinnae, and roots (Youngstrom et al., 2019). Knockdown of *CrWUL*, which also belongs to the *WOX* family, also reduces the number of roots and residing phloem cells (Youngstrom et al., 2022). Another *WOX* gene probably accounts for formation of nearly all cell types in roots (Nardmann and Werr, 2012). *LEAFY* (*LFY*) maintains apical stem cell activity during shoot development in *C. richardii* (Plackett et al., 2018; Rodríguez-Pelayo et al., 2022). In this study, we conducted a spatial transcriptomic analysis of sporophyte development in *C. richardii*, founding transcription factors (TFs) that may be crucial to growth and development of this water fern, and ribosomal protein (RP) gene heterogeneous expression that can translationally regulate gene activity, leaving an open question of relationship between TFs and ribosomal biology.

## Materials and methods

### Plant materials

The water fern *C. richardii* strain Hnn was used in this study. Spores were germinated, and gametophytes were cultured axenically in liquid Murashige and Skoog (MS) medium supplemented with 2% sucrose. The cultures were maintained at 25°C under a 16/8-h light/dark cycle to facilitate gametophyte fertilization and sporophyte formation. The resulting sporophytes were subsequently transferred to soil-containing pots. This cultivation protocol was performed as previously described (Xiao and Li, 2024).

### Cryo sectioning

A sporophyte at the vegetative growth stage with three fully expanded leaves was dissected to isolate the central shoot tip. The tip was immediately embedded in optimal cutting temperature (OCT) compound (Sakura Finetek Europe B.V.) and frozen on dry ice. Serial sections were cut at a thickness of 20 μm, and the resulting tissue sections were anchored onto glass slides and fixed with 100% methanol. Subsequently, the sections were stained with toluidine blue for histological analysis.

### Construction and sequencing of libraries

Spatial transcriptomic analysis was performed using the BMKMANU S1000 platform (BioMarker, Beijing). This platform employs a high-density spot array of over 2 million spots (diameter < 2.5 μm, 5 μm center-to-center distance) within a 6.8 mm² area, where poly(T)VN oligonucleotides immobilized poly-adenylated RNA. Double-stranded cDNA was synthesized on the beads, incorporating the spatial barcode, unique molecular identifiers (UMIs), and the sample index. The resulting cDNA library was then fragmented via enzymatic digestion and sequenced on an Illumina NovaSeq platform using the PE150 strategy. DNA contamination and low-quality reads were filtered using SOAPnuke v1.4.0 (Chen et al., 2018). Clean sequencing reads were mapped to genes using Bowtie2 v2.2.5 (Langmead and Salzberg, 2012), which provided a basis for estimating gene expression levels. The raw sequencing data, available under GEO accession GSE307951, were aligned to the GCA_020310875.1 reference genome (Marchant et al., 2022).

### Gene expression matrix analysis

The spatial gene expression matrix was constructed using BSTMatrix (BioMarker) at eight different resolution levels, ranging from 5 μm (Level 1), which resolves individual spots, to 100 μm (Level 13), which aggregates 469 spots into a single super-spot. For downstream analysis, we primarily used data from Level 7 (50 μm resolution), which yielded 3,003 super-spots. Data normalization, spot clustering, and the identification of differentially expressed genes (DEGs) were performed using Seurat v5 (Butler et al., 2018). DEGs were defined by thresholds of |log2Fold Change| ≥ 0.5 and a false discovery rate (FDR) < 0.05. Unique molecular identifier (UMI) counts were normalized with a negative binomial generalized linear model, and regularized Pearson residuals were subsequently calculated for accounting for gene expression levels. Z-scores were further acquired for heatmap presentation. The cluster results were visualized using BSTViewer V1.42 (BioMarker). Protein sequences were queried against the NR, Pfam, Swiss-Prot, and TrEMBL databases using BLAST^®^ for functional annotation. Gene set enrichment analysis was subsequently conducted using clusterProfiler v3.18.1. TFs were identified and classified based on the Plant Transcription Factor Database (https://planttfdb.gao-lab.org/index.php).

### Identification of RP genes

We used a set of 81 representative RP sequences from *A. thaliana* and *Oryza sativa* as queries (Lan et al., 2022; Scarpin et al., 2023) to search against the *C. richardii* proteome (Marchant et al., 2022). The putative homologs displaying an e-value smaller than 1e-004 were subsequently aligned with the query sequences using the ClustalW2 program (Thompson et al., 1994). The resulting multiple sequence alignments were manually inspected mainly in terms of gene sequence length to confirm the *C. richardii* RP gene identity.

### RNA *in situ* hybridization

The shoot tips were fixed in FAA fixative at 4°C for approximately 12 h, followed by dehydration, clearing, and paraffin embedding, as we previously conducted (Xiang and Li, 2024; Xiao and Li, 2024). Sections were cut at a thickness of 8 μm and mounted on poly-lysine-coated slides for subsequent *in situ* hybridization. Gene-specific fragments for probes were amplified using the following primers: 38G026900 using 10g044-f: 5’-TACGGTCAAAGGGCAGTTCT and 10g044-r: 5’-TCAGCTCCTCCCCATTGATC, 23G070600 using 23g070-f: 5’-CCGCCTAAATTGGATCCGTC and 23g070-r: 5’-CATCAACAGTGCACCCTACG, and 10G044300 using 10g044-f: 5’-TACGGTCAAAGGGCAGTTCT and 10g044-r0: 5’-TGGACCAGCTGATTCCCAAT. The PCR products were cloned into the T-easy vector (Promega). Then, linearized DNA templates were used for *in vitro* transcription with T7 or SP6 RNA polymerase to synthesize digoxigenin-labeled antisense RNA probes. These probes were hydrolyzed to an average length of approximately 200 nucleotides. The hydrated sections were treated with proteinase K (2 μg mL^-1^) from *Trichosporon cutaneum* for 30 min at 37°C before hybridization. The probes were applied to the sections and incubated at 50°C for approximately 10 h. After hybridization, stringent washes were performed using 0.2× SSC buffer at 55°C for 3 h. The anti-digoxigenin antibody (Roche) was diluted 1∶1000 in TBS buffer containing 0.3% Triton X-100, and incubation was performed in the same buffer for 2 h at room temperature. Subsequently, slides were washed using the TBS buffer containing Triton X-100 that was retained from the previous procedure for 15 min, followed by three 15-min washes in TBS buffer without Triton X-100. The BCIP/NBT solution (Roche) was diluted 1∶50 in TNM buffer (pH 9.5), and the subsequent incubation with sections lasted for about 18 h at room temperature in darkness. Finally, sections were air-dried and mounted using a xylene-based medium, and the blue-purple precipitate was observed using a Leica DM3000 light microscope.

## Results

### Tissue allocation of spot clusters

**Figure 1:**
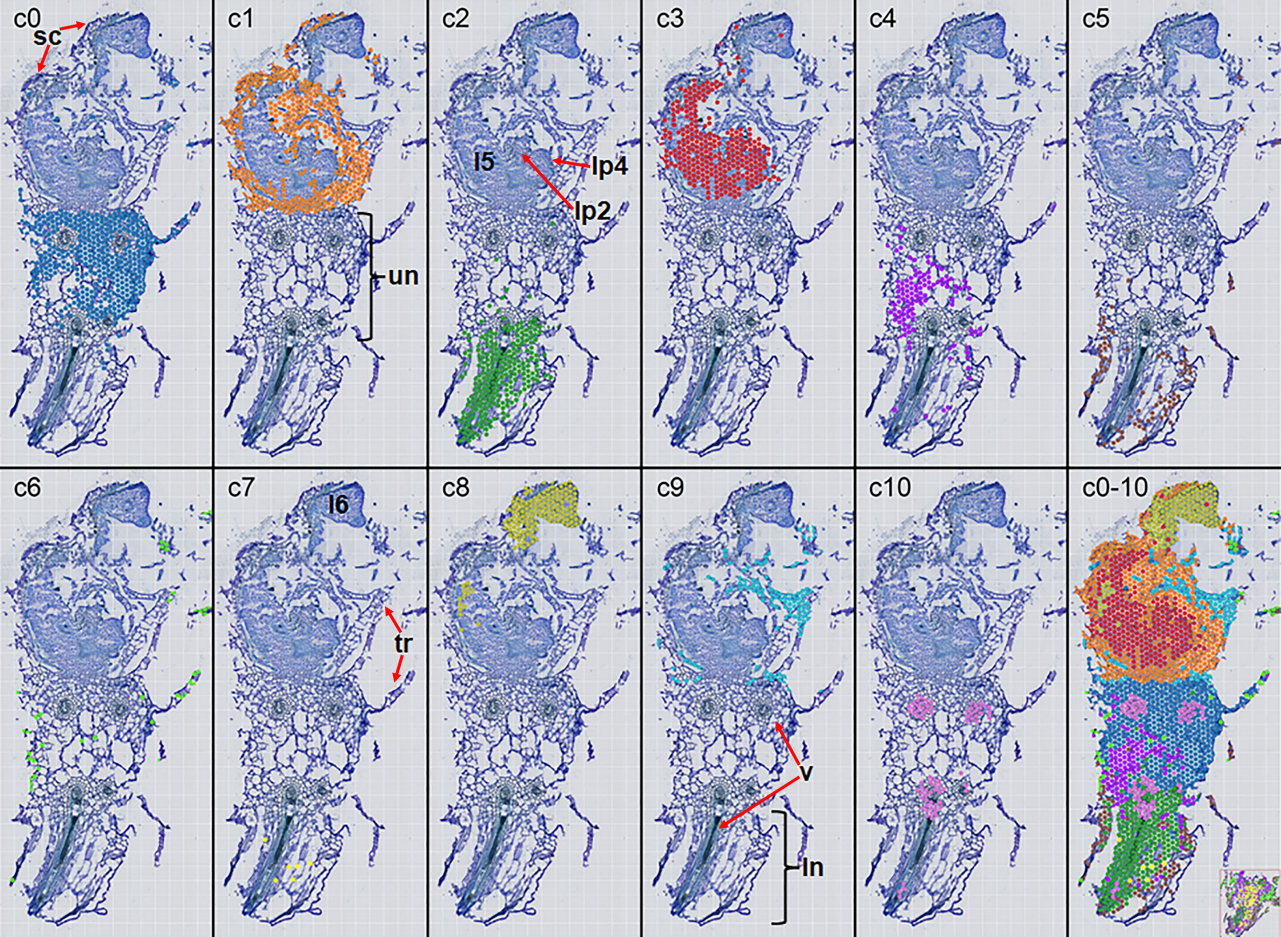
Tissue allocation of spot clusters. Names of clusters were indicated in the upper left corner of pictures, while tissues occupied by clusters were indicated by certain colors, etc. c0 by gray blue. The c0–10 picture was the merged one, and at its lower right corner was another merged picture of a different section. sc, scales; un, the upper node; l5, the 5^th^ leaf; lp2, the 2^nd^ leaf primordium; lp4, the 4^th^ leaf primordium; tr, trichomes; v, vasculature; ln, the lower node.

Spatial transcriptomic analysis of the shoot tip of a *C. richardii* vegetative sporophyte yielded high-quality data, free of ambiguous bases (**Supplementary Figure S1**; **Table 1**). The number of detected genes remained stable across a total of eight levels of resolution, ranging from 24,031 in Level 3 to 24,071 in level 13. At Level 7 resolution (50 μm), the dataset comprised 3,003 super-spots (amalgamating 127 physical spots), with a median of 367 molecules and 280 genes per super-spot, culminating in the identification of 24,048 genes. Unsupervised clustering of this expression matrix (parameters: level 7, 100 genes, 100 spots, resolution 1.5) defined 11 distinct spot clusters (**Figure 1**). These clusters revealed a complex spatial organization: cluster c0 was predominantly localized to the proximal part of the leaf petiole affiliated to the upper node of the stem, co-occurring with the more restricted c4 cluster without a sharp histological boundary. Cluster c10 was specifically enriched in the vasculature that led to roots. The lower node of the stem was characterized by a central c2 domain existing in the vasculature running through the stem. There was a peripheral c5 domain and a central c7 domain in the stem. Finally, cluster c6 marked the plant surface and was associated with trichomes that typically aerially project away from the plant body. Remarkably, these clusters were similarly distributed in an independent section which pitifully, was off-center (**Supplementary Figure S2**).

**Table 1:** Parameters of the spatial transcriptome generated.

Properties	Quantities
Read number	634544568
Barcode number	563332977
UMI number	629654452
Gene number	24031~24071
Mapped to the genome	93.14%
Mapped to the exon	87.90%
Sequencing saturation	98.60%

The remaining clusters were associated with the developing leaves. Cluster c1 encapsulated two young leaves, likely representing the domain of scales that differ from general trichomes in terms of proximity to the plant body and the structural morphology. In contrast, the sparser c9 cluster was distributed in the domain of general trichomes. Cluster c3 was primarily localized to the proximal part of the 5^th^ young leaf, the presumptive 2^nd^ and 4^th^ leaf primordia and possibly the SAM. Finally, cluster c8 was specific to the distal part of the 6^th^ young leaf. Collectively, these 11 clusters provided comprehensive coverage of all tissue domains within the section, leaving no significant areas unaccounted for.

### Identification of TFs

**Figure 2:**
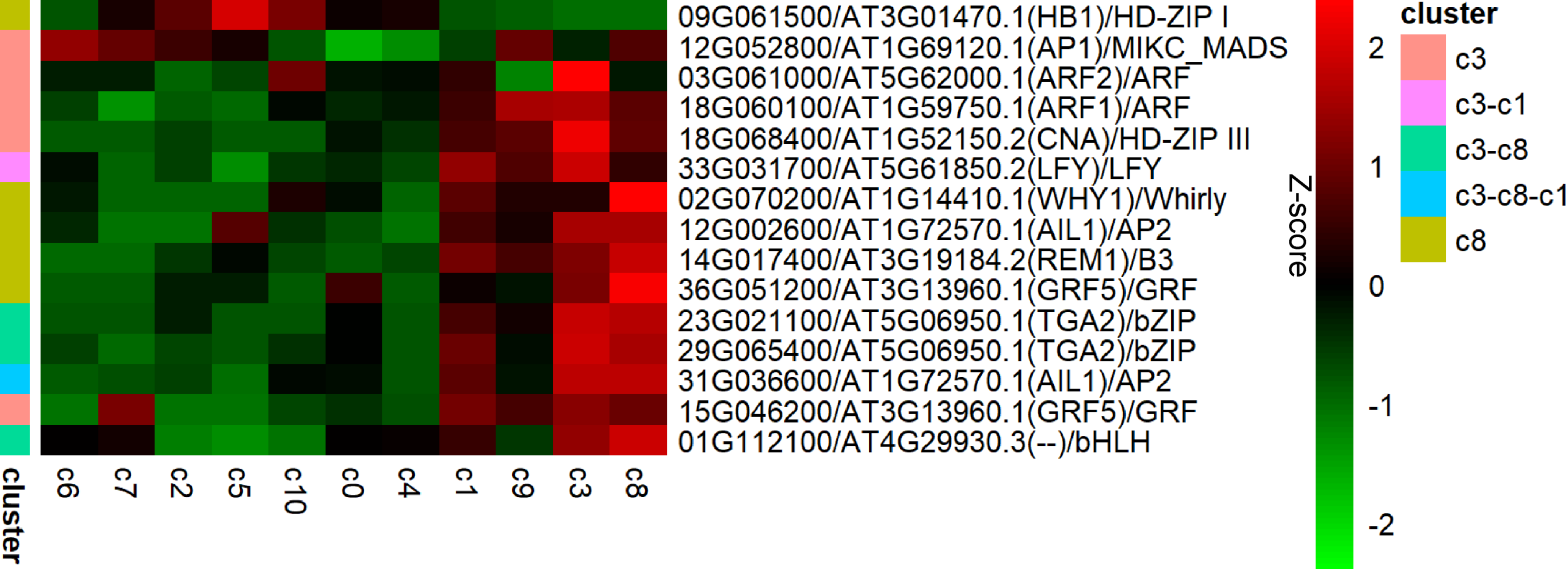
TFs among feature genes. Rows of expression levels observed in all spot clusters were followed with gene loci of these TFs, gene loci and gene names of best hits, and the affiliated gene families.

Comparative analysis identified 1,187 differentially expressed genes (DEGs) that served as marker genes for the spot clusters, derived from a pool of 2,692 feature genes and 4,892 SVGs. Among these markers, we identified 14 TFs with high sequence similarity to *A. thaliana* homologs (E-values: 6e-027 to 0) (**Figure 2**). These TFs were exclusively assigned to the leaf-associated clusters c1, c3, and c8, with no TF markers found for the eight clusters residing in stem tissues. These TFs belonged to 11 families, with two members representing the *ARF*, *GRF*, *AP2*, and *bZIP* families, respectively. Several different TFs hit the same *A. thaliana* homologs, indicating a close relationship between them. For example, 36G051200 and 15G046200 both hit *GRF5*, while 23G021100 and 29G065400 both hit *TGA2*. A notable example is 09G061500, which marked cluster c8 for low expression in comparison of c8 with all other clusters. When clustering was confined to c3 and c8 domains, c3 domain was divided into three sub-domains; when a certain sub-domain was compared with other domain/sub-domains, 12G052800 was also identified as a marker, of course related to c3, for low expression. Functional enrichment revealed that 7 of these TFs are putatively involved in shoot system development (GO:0048367) and 5 in phyllome development (GO:0048827), underscoring their role in leaf morphogenesis.

In addition to the 14 cluster-specific TF markers, 26 other TFs were identified that were uniformly expressed across all clusters but still showed significant functional enrichment for the shoot system and phyllome development (**Supplementary Table S1**). Furthermore, an additional 81 TFs were defined as SVGs, and this group was also significantly enriched for the same developmental processes, reinforcing the central role of spatially regulated transcription in shoot and leaf development (**Supplementary Table S1**).

### GO annotation of the differentially expressed genes

**Figure 3:**
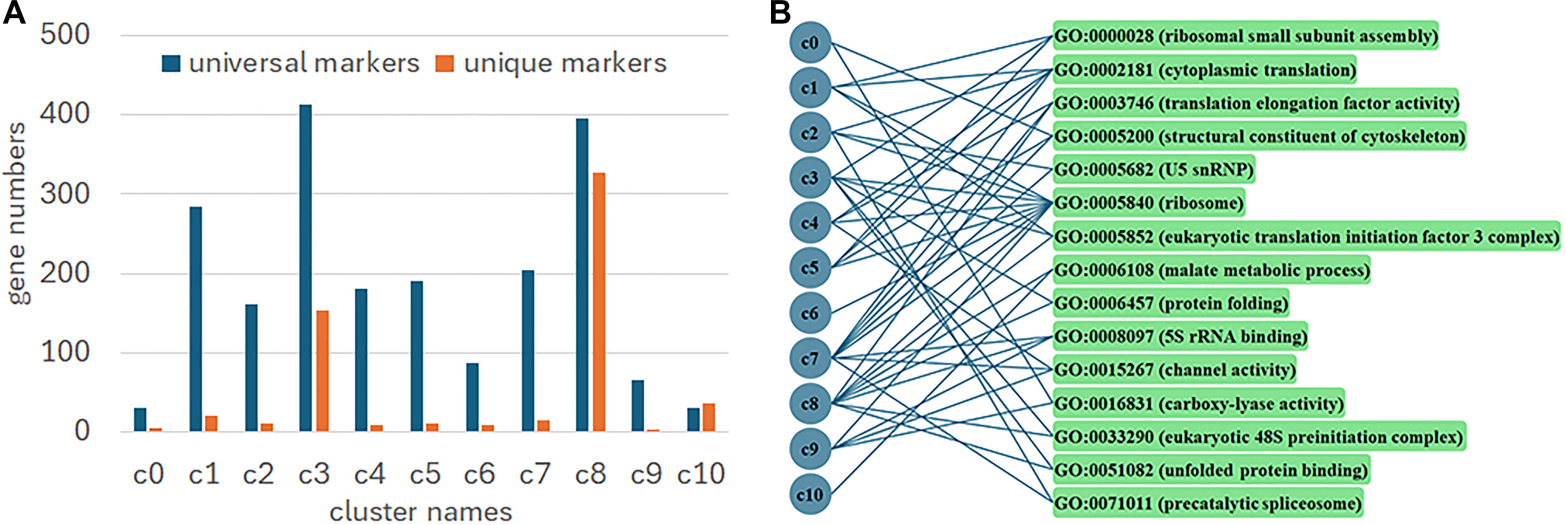
Numbers of markers among clusters. **(A)** Proportions of universal and unique markers in every cluster; **(B)** Distribution of universal GO terms among clusters.

Analysis of cluster-specific marker genes revealed substantial variation in their proportion across clusters, ranging from low (e.g., c1 at 6.89%) to very high (e.g., c10 at 54.55%) (**Figure 3A**). Cluster c0 markers were uniquely associated with defense response (GO:0006952) and xyloglucosyl transferase activity (GO:0016762). Clusters c3 and c7 were enriched for unique terms including DNA helicase activity (GO:0003678) and sterol biosynthesis (GO:0016126), and GTPase activity (GO:0003924), respectively. Cluster c8, associated with young leaves, displayed a unique chloroplast-related signature (e.g., chlorophyll binding (GO:0016168), thylakoid (GO:0009579), and chloroplast stroma (GO:0009570)). In contrast, the trichome-related cluster c9 was uniquely defined by cell wall (GO:0005618), pyridoxal phosphate binding (GO:0030170), and toxin activity (GO:0090729). No unique GO terms were identified for clusters c1, c2, c4, c5, c6, and c10.

Beyond cluster-specific functions, marker gene analysis revealed universal GO terms shared across multiple clusters (**Figure 3B**). The number of these shared terms varied from one in c10 to eight in c7 and c8. Most terms were confined to two or three clusters, regardless of the tissue type. Ribosome-related functions were exceptionally pervasive: the term “ribosome” (GO:0005840) was enriched in nine clusters, and “cytoplasmic translation” (GO:0002181) was enriched in five. This prevalence was driven by RP genes, which constituted 61.62% of all marker genes used for GO term determination, indicating their widespread differential expression across diverse tissue domains.

### Characterization of RP genes

We identified a near-complete repertoire of 80 RP types in *C. richardii*, compared with 81 in *A. thaliana* (**Supplementary Table S2**). The missing type, eL41, is notably short (~25 aa) and is also absent in the annotated genomes of *Azolla filiculoides* (v1.2) (Li et al., 2018). The eL41 has been renamed as eS32 for tighter association with the SSU of ribosomes (Hassan et al., 2025), and it is generally not easy to bioinformatically find despite being relatively dispensable and even missing in some archaeans (Lecompte et al., 2002). Most RP types were related to multiple genes, and thus there were a total of 218 RP genes. Expression analysis revealed that 170 of these genes (spanning all 80 types) were differentially expressed between clusters. An additional 12 genes (from 11 types) were uniformly expressed, whereas the remaining 36 genes (from 22 types) were not detected. At least one gene was expressed for every RP type.

**Figure 4:**
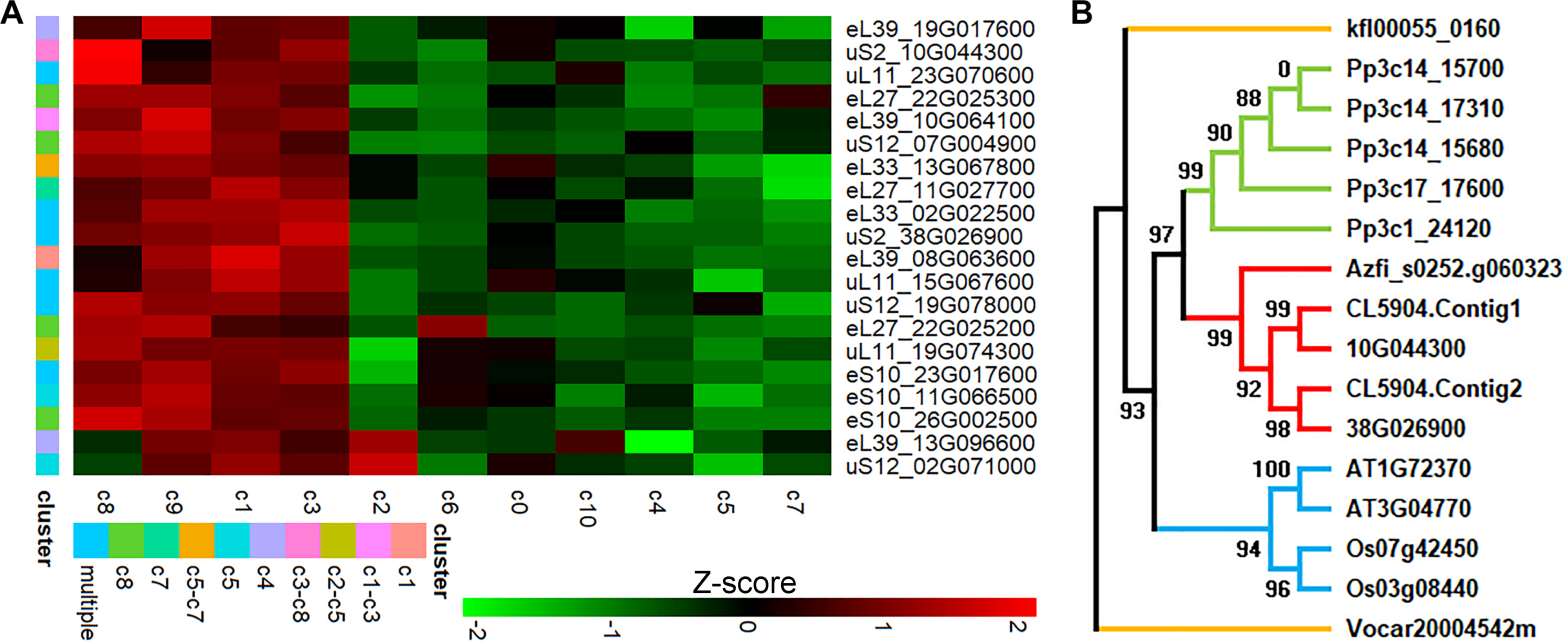
Characterization of RP genes. **(A)** Expression levels of representative genes across spot clusters. Genes were possibly differentially expressed to mark a single (c1, c4, c5, c7, and c8), two (c1-c3, c2-c5, c3-c8, c5-c7), or multiple spot clusters (three or more clusters); **(B)** A maximum-likelihood tree of representative uS2 genes. The number of bootstrap replicates was 1000. The brown, green, blue, and red branches indicated the alga, bryophyte, angiosperm, and fern genes, respectively. AT, *Arabidopsis thaliana*; Os, *Oryza sativa*; Pp, *Physcomitrium patens*; Kfl, *Klebsormidium flaccidum*; Vocar, *Volvox carteri*; Azfi, *Azolla filiculoides*; CL5904.Contig1/2 came from *Ceratopteris pteridoides*, and 10G044300 and 38G026900 came from *C richardii*.

Many RP genes served as specific markers for individual spot clusters, and there were 11 such genes for the leaf-associated cluster c8, which was the most prominent example. However, most differentially expressed RP genes simultaneously marked multiple clusters. A prominent example is 19G078000 belonging to the uS12 family, marking seven different clusters (c0, c1, c2, c3, c7, c8, c9) (**Figure 4A**). Especially, individual members from the same families had exhibited different spatial expression programs since they were not clustered together since expression levels observed in every tissue domain. For instance, while 19G078000 had a distinct expression level in up to seven clusters, other uS12 paralogs like 02G071000 and 07G004900 were just differentially expressed in c5 and c8, respectively, because they were separated in clustering based on expression levels. Clustering also revealed expression divergence among paralogs for the eL27 and eL39 families. Particularly, paralogs that even marked the same cluster (e.g., the two eL39 genes in c4) could be divergent in the global expression pattern. This widespread differential expression among paralogs was observed in all 80 RP types, occurring to 51 types (63.75%) with all expressed genes diverging in the global expression pattern. For example, there were only two genes for the uS2 family in *C. richardii*, with 38G026900 being a universal marker for six clusters (c1-c5, c7), while 10g044300 being a marker restricted to only c3 and c8 (**Figures 4A, B**).

### *In situ* hybridization of the three RP genes

**Figure 5:**
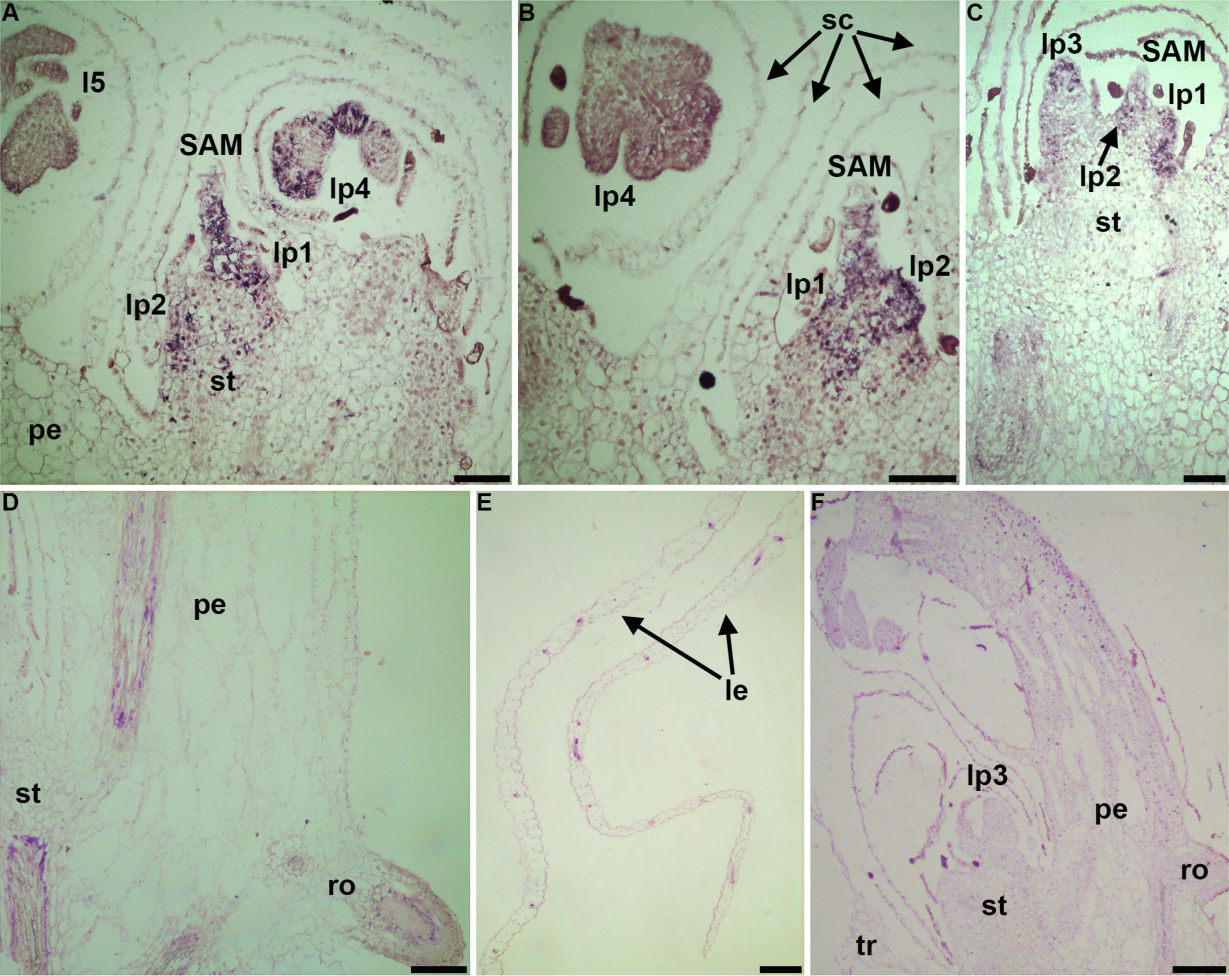
RNA tissue *in situ* hybridization of the RP gene 23g070600. **(A)** Expression in the SAM, leaf primordia, pinna primordia, and the vasculature in the stem; **(B)** Expression in the SAM, leaf primordia, and pinna primordia in the fourth leaf primordium; **(C)** Expression in the SAM and leaf primordia; **(D)** Expression in the vasculature in the petiole, stem, and root; **(E)** Expression in the vasculature in the leaf blade; **(F)** Sense probe hybridization as a control.

The uL11 family member 23g070600, a marker for multiple clusters (c1, c3, c4, c6, c7, c8), exemplifies the divergence of expression among RP paralogs. It has two paralogs: 19g074300 (~99% nucleotide identity) which marks c2 and c5, and 15g067600 (~82% identity) which marks c1, c2, and c5. 23g070600 was expressed in the SAM, the 1^st^and 2^nd^ leaf primordia, and the pinna primordia in the 4^th^ leaf primordium and the 5^th^ young leaf (**Figures 5A, B**). At the same time, scales were not obviously stained by gene probe hybridization This gene was also expressed in the 3^rd^ leaf primordium (**Figure 5C**). The vasculature was stained in the petiole, stem, and root (**Figures 5C, D**), and it was also expressed in the vasculature of the blade in young leaves (**Figure 5E**). As a control, the sense probe produced no signal in various regions, including the third leaf primordium (**Figure 5F**).

## Discussion

### Tissue domain implications of spot clusters

Cluster c3 concerns the assumptive 5^th^ leaf, the younger leaf primordia, and probably the SAM, where there is an alternate phyllotaxis (Sun and Li, 2020). In contrast, cluster c8 covers the more advanced 6^th^ leaf, thus providing an opportunity for studying genes involved in leaf developmental stage other than determinacy and size in *C. richardii* (Cruz et al., 2020; Xiang and Li, 2024). Surrounding the leaf primordium, clusters c1 and c9 likely represent early scales and later scales called trichomes here, respectively (Conway and Di Stilio, 2020). The proximal part of the leaf petiole affiliated to the upper node of the stem is defined by clusters c0, c4, c6, and c10, whereas the stem of the lower node is featured by clusters c2, c5, and c7, thus unveiling their characteristics more. Clusters c10 and c2 are nearly specific to the vasculature leading to roots and that running though the stem, respectively, seeming complementarily distributed and lacking in young leaves. Cluster c6 is distributed in the trichome domain located at the stem, which contrasts with c9 that occurs to leaf trichomes. Clusters c5 and c7 occupy the peripheral and the central parts of the stem, respectively, whereas clusters c0 and c4 divide the proximal part of the leaf petiole without obvious preference to certain tissues. Therefore, there are novel tissue domains that have not been explored in *C. richardii*.

### TFs probably involved in leaf development

TFs are generally the central hub of gene networks in plants. In our study, differentially expressed TFs were mostly linked to leaf-related domains, suggesting for them a role in development of young leaves, leaf primordia, the SAM, and possibly sporangia which can be seen early as on the 4^th^ leaf primordium. Furthermore, these TF are very similar in the protein sequence to several angiosperm genes key to development of equivalent tissues and organs. For instance, *ARF1* and *ARF2* can bind to the *GRF5* promoter and thus regulate leaf senescence (Beltramino et al., 2021), and the *why1* mutant has an early-senescence phenotype in leaves (Miao et al., 2013). There is positive feedback between *LFY* and *AP1* when the SAM ceases to produce leaves to generate flowers (Liljegren et al., 1999), whereas *CNA* can promote stem cell differentiation in the SAM (Green et al., 2005) and *AIL*s play a role in meristem development and organ initiation and outgrowth (Horstman et al., 2014). Finally, 01G112100 is on one hand positioned within leaf-related domains, and it is on the other hand close to *AMS* and *JAM1* involved in microspore development (Qian et al., 2024), leaving an open question since the sampled plants are at stage S3 during vegetative growth and sporangia cannot emerge until the reproductive stage S6 in *C. richardii* (Conway and Di Stilio, 2020). These conservations might suggest a common genetic framework for leaf development predates divergence between ferns and seed plants (Tomescu, 2009; Vasco et al., 2016), even if the precise functional mechanism has possibly changed during evolution (Sun and Li, 2020).

### Functional implications of spot clusters

Cluster c3 covers not only young leaves but also probably leaf primordia and even the SAM, where mitosis actively occurs, thus being enriched in genes related to DNA helicase and sterol biosynthesis critical to cell formation (Boutté and Grebe, 2009). In contrast, cluster c8, which is associated with a more developed young leaf, is active in photosynthesis and chloroplast-related functions. Cluster c0 is located at the proximal part of the leaf petiole affiliated to the upper node of the stem—a region poised for elongation and interaction with the environment, thus being enhanced in terms of defense response and xyloglucosyl transferase activity. The latter function, which is involved in cell wall loosening, also can mitigate biotic and abiotic stresses (Ishida and Yokoyama, 2022). Thus, these spatial transcriptomic patterns enable assignment of specific biological functions to tissue domains in *C. richardii*.

While it is possible for a certain GO term to be predicted in multiple spatial clusters because of a handful of genes that belong to the same pathway or network being differentially expressed, the pervasive enrichment of ribosome-related functions across nearly all clusters is remarkable. This phenomenon has been partly observed in other plant spatial transcriptomes (Liu et al., 2022; Lv et al., 2024). Thus, it is an open question whether and how differential expressions of ribosome-related genes play a role in specifying plant tissues or parts.

### Heterogeneous expression of RP genes

RP genes have not all been simultaneously expressed in a spatial transcriptome in *C. richardii*, thus revealing for them a heterogeneous expression. At the same time, RP genes are either uniformly or differentially expressed across tissue domains. Especially, paralogous RP genes can also be mutually different for no expression, uniform expression, or differential expression. Furthermore, Paralogous RP genes display either single-cluster specificity, or complex multi-cluster specificity. This pervasive heterogenous expression across the RP gene repertoire suggests a mechanism for generating ribosomes with potentially specialized functions in different tissues. Certainly, this finding is consistent with tissue-specific RP transcript populations in *Brassica napus* (Whittle and Krochko, 2009), and cell-specific expression of RP genes in animals (Guimaraes and Zavolan, 2016). Moreover, the presence of ubiquitous and specialized paralogs (Kearse et al., 2011), compensatory expression (Chaillou et al., 2016), and autogenous regulation (O’Leary et al., 2013), have been established for heterogeneous expression of RP genes. Finally, microarray data indicates that a subset of RP genes in *A. thaliana* exhibits a different and specific expression pattern in response to environmental stress (Sormani et al., 2011). We propose that this transcriptional heterogeneity may underpin formation of the identified numerous tissue domains in *C. richardii*. RP genes are highly expressed, and thus they can be statistically identified as differentially expressed genes even though there is only subtle difference in mRNA abundance for them, which possibly leads to their heterogeneous expression in this spatial transcriptomic technique which has a low read depth.

### Tissue-specific expression of RP genes

RP genes are specifically expressed in the SAM, leaf primordia, and the vascular, where many cells are pluripotent and thus mitosis is active (Atta et al., 2009). This observation is, therefore, consistent with association of RP genes with cell division and differentiation in *A. thaliana* (Weijers et al., 2001). Furthermore, ribosome complexes are so stable that RP genes are only transcribed in newly generated cells (Barna et al., 2022). It is interesting to explore whether tissue-specific transcribing of RP genes has resulted in ribosome abundance and translational activity differing between the germline and the somatic tissues, or even between the distal and the proximal germline in *Caenorhabditis elegans* (Somers et al., 2022). Tissue-specific expression of RP genes can accommodate their paralog-specific expression, providing rooms for generation of biochemically specialized ribosomes (Park et al., 2024), or homeostasis affected ribosomes (Martinez et al., 2025). The functionally altered ribosomes may preferentially translate certain mRNAs (Martinez-Seidel et al., 2020; Li et al., 2023), thus causing severe developmental defects when specific RP genes are disrupted (Williams and Sussex, 1995; Degenhardt and Bonham-Smith, 2008; Horiguchi et al., 2011; Xiong et al., 2020).

## Data Availability

The datasets presented in this study can be found in online repositories. The names of the repository/repositories and accession number(s) can be found below: https://www.ncbi.nlm.nih.gov/, GSE307951.

## Supplementary material

Supplementary Figure 1 The sampled plant and sectioned specimen. The dissected specimen was explained with red and black arrows indicating transparent and greenish scales, respectively. Additionally, the asterisk marked the part presumptively consisting of the SAM, and the 1^st^ to the 4^th^ leaf primordia.

Supplementary Figure 2 The second section with or without superimposed cluster dots.

Supplementary Table 1 TFs identified as feature genes excluding marker genes and SVGs excluding feature genes in this spatial transcriptome.

Supplementary Table 2 All RP genes identified in *C. richardii*. Numbers were regularized Pearson residuals.
