# Supplementary figures and images for: Spatial regulation of ribosomal protein gene expression revealed by spatial transcriptomic analysis in the water fern Ceratopteris richardii
Source: Front Plant Sci. 2026 Jan 19;16:1728120. doi: 10.3389/fpls.2025.1728120 (PMC12862073; doi:10.3389/fpls.2025.1728120)

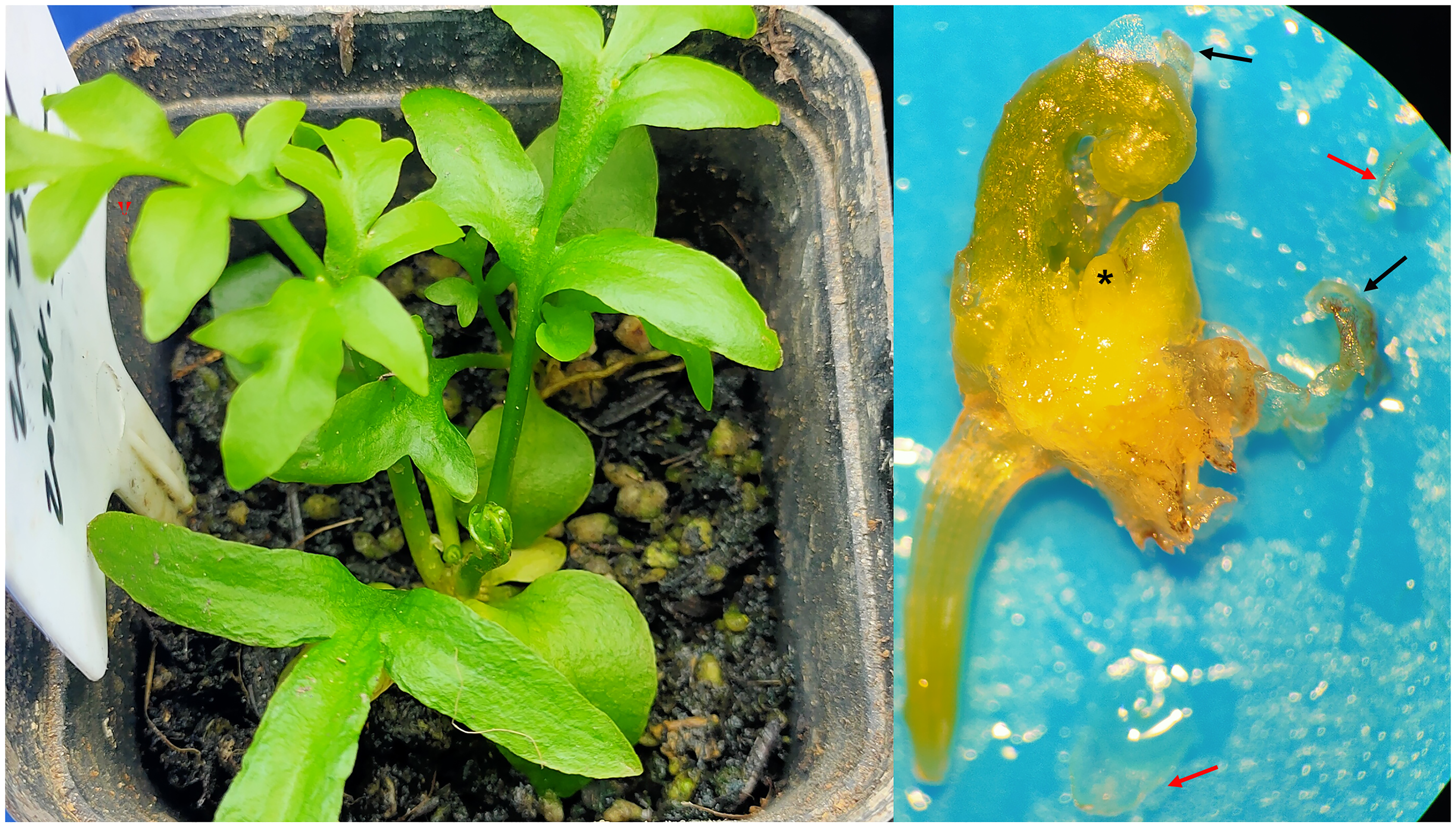

Supplement: Supplementary Figure 1 — The sampled plant and sectioned specimen. The dissected specimen was explained with red and black arrows indicating transparent and greenish scales, respectively. Additionally, the asterisk marked the part presumptively consisting of the SAM, and the 1st to the 4th leaf primordia. [file Image1.tif]

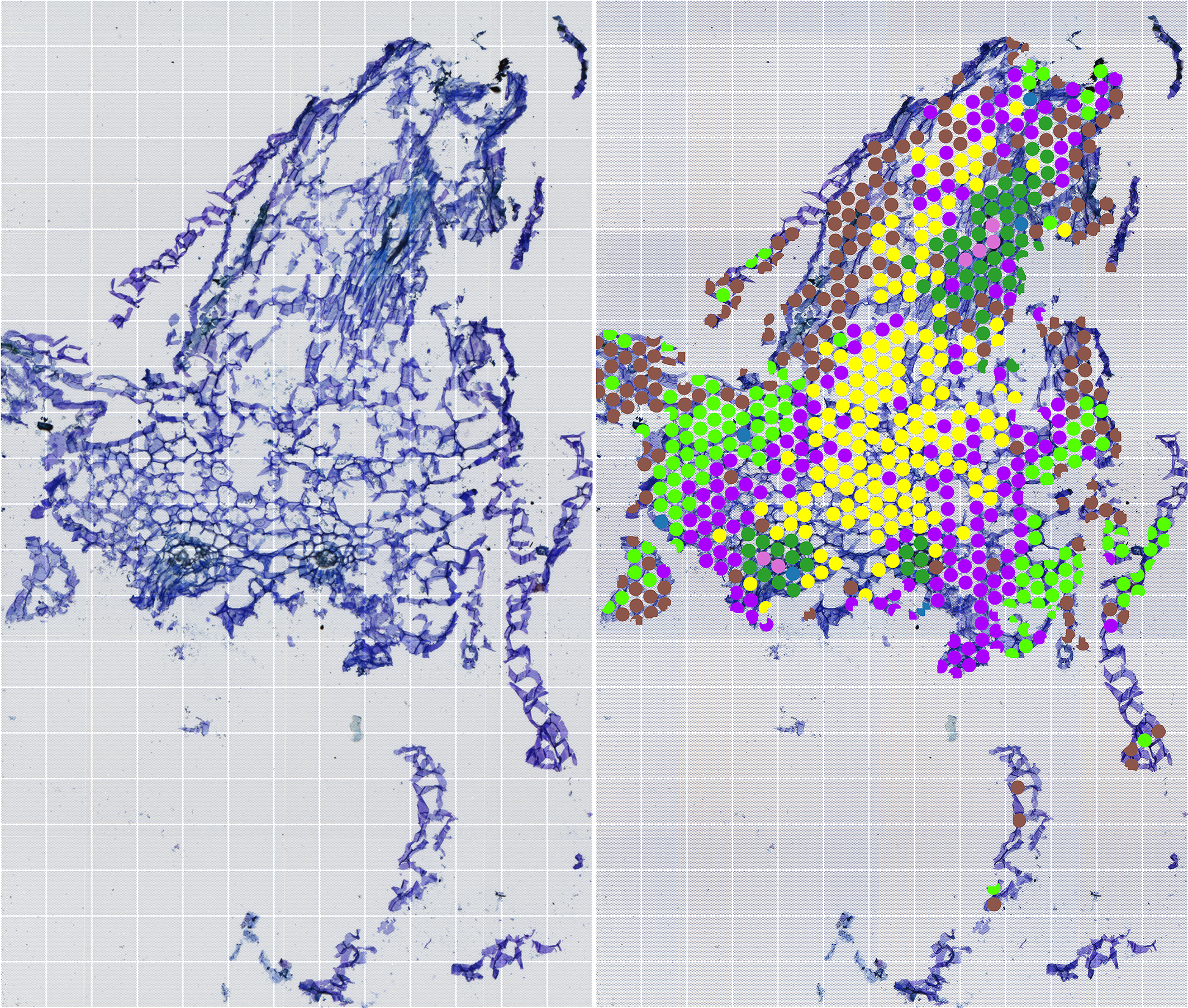

Supplement: Supplementary Figure 2 — The second section with or without superimposed cluster dots. [file Image2.tif]
